# Micromechanical evaluation of DP1000-GI dual-phase high-strength steel resistance spot weld

**DOI:** 10.1007/s10853-018-2886-z

**Published:** 2018-09-11

**Authors:** A. Chabok, E. Galinmoghaddam, J. T. M. De Hosson, Y. T. Pei

**Affiliations:** 1 Department of Advanced Production Engineering, Engineering and Technology Institute Groningen, University of Groningen, Nijenborgh 4, 9747 AG Groningen, The Netherlands; 2 Department of Applied Physics, Zernike Institute for Advanced Materials, University of Groningen, Nijenborgh 4, 9747 AG Groningen, The Netherlands

## Abstract

In situ micro-cantilever bending tests were carried out on resistance spot welded DP1000-GI dual-phase high-strength steel in order to derive the mechanical response of the welds. Notched micro-cantilevers were milled using focused ion beam milling at the base metal, inter-critical, fine-grained and coarse-grained heat affected zones, and fusion zone. It was shown that due to large plastic yielding, linear-elastic fracture mechanics are inapplicable. To evaluate the fracture toughness of different weld zones, cyclic loading was applied to track the crack size and the conditional fracture toughness of weld zones was measured using crack tip opening displacement and *J*-integral methods. It was found that micro-cantilever bending method provides insight to the fracture toughness and local mechanical response of different weld zones. The results obtained can be used to make an accurate correlation between resistance spot welding process, microstructure and mechanical response of DP1000-GI dual-phase high-strength steel welds.

## Introduction

Advanced high strength steels (AHSS), including dual-phase (DP), belong to a new generation of key materials in the design and production of car body structures. Their use has been steadily increasing over recent years in automotive industry. This is attributed to the advantages of AHSS grades offering higher strength and ductility that enable decreasing the vehicle weight for improved fuel economy and reduced impact to the environment while improving crash energy absorption for better protection.

Resistance spot welding (RSW) is the predominant joining technique in automobile body production with a typical vehicle containing 4000–5000 spot welds. Therefore, the safety of vehicles is to a large extent determined by the properties of resistance spot welds that assemble all steel components together. While RSW technique is well-established for the traditional mild steels, AHSS are known to be more susceptible to weld metal failure of resistance spot welds [1]. AHSS often suffer from degraded fracture resistance and rather low toughness of the welds. This is attributed to higher content of alloying element of AHSS that leads to the formation of brittle phases and microsegregation phenomena within the fusion zone (FZ) of the spot weld. A resistance spot weld usually constitutes of complex microstructure gradients with a variety of mechanical responses in a confined space. The failure mode and failure mechanism of spot welds depend on the complex interplay between the local mechanical properties of the FZ, heat affected zone (HAZ), base metal (BM) and the final stress states in the weld [2].

Different models have been proposed to derive the critical weld nugget size and to predict strength of resistance spot welds [3–5]. They are mainly based on local mechanical properties of resistance spot weld such as fracture toughness, yield strength and ductility of different zones. It was already shown that the fracture toughness of the weld is one of the most effective factors determining the mechanical properties of resistance spot welds [6, 7]. However, considering the small size of spot welds (typically 5–7 mm) and the size of heat affected zones (ranging from 0.1 to 0.7 mm), local mechanical characterization of spot welds necessitates unique experimental approaches. Tong et al. [8] used miniaturized tensile bars with a length of 3 mm to measure tensile properties of the welds. However, a simulated microstructure of HAZ was used, as it was impossible to cut such a sample from its narrow area. Also, it was impossible to analyze the mechanical performance of fine-grained (FG), coarse grained (CG) and inter-critical (IC) heat affected zones separately. Nanoindentation is also routinely used to investigate the mechanical properties of different zones [9]. However, it cannot be used to evaluate the fracture toughness of ductile phases that are formed during RSW, as it is based on the length of cracks emanating from the residual indentation impression.

Recently, fracture analysis using notched micro-cantilevers made by focused ion beam (FIB) milling was developed. Advent of in situ electron microscopy-based fracture instruments has provided a solid base for this novel approach. Using this method, stress intensity factor was successfully measured for NiAl single crystals [10], WC-based coatings [11], Si single crystal [12] and zirconia [13]. While most of the investigated materials showed brittle behavior even at micro-scale, Wurster et al. [14] applied the *J*-integral and crack tip opening displacement methods successfully to critically evaluate the fracture toughness of tungsten single crystal which failed in a ductile manner. Costion et al. [15] measured the fracture toughness of acicular ferrite and upper bainite. They found that despite different microstructural characteristics of two phases, their mechanical responses at microscale are quite similar.

The response of resistance spot welds to mechanical loading is significantly different from that of the base metals. That is so because of the microstructure/property gradients formed in the FZ and HAZ [16], as well as due to the geometrical constraints of spot welding. Strength and hardness mismatch among the FZ, HAZ and BM create stress concentrations in the microstructural zone of the lowest strength or hardness under deformation. In order to predict the mechanical performance and failure of spot welds, therefore, the gradients and mechanical properties must be determined at a microstructural level.

To the best of our knowledge, there is no literature reporting the strength and fracture toughness of different microstructural zones in AHSS spot welds. This is largely attributed to the fact that direct measurement of mechanical properties of different regions of spot weld is hardly possible without appropriate means such as FIB cutting of micro-specimens and in situ testing devices. The present work aims at studying the fracture behavior of different weld zones at micrometer-scale using FIB made notched micro-cantilevers.

## Experimental

DP 1000 AHSS 1.5-mm-thick plates were resistance spot welded using a 1000 Hz MFDC pedestal welding machine with constant current regulation and constant load of 4.5 kN. Welding electrodes (F1 16-20-5.5) and weld scheme were taken from the VDEh SEP1220-2 welding standard [17].

After sectioning of the weld through the center, its cross section was ground and polished mechanically, followed by a chemical–mechanical polishing with a mixture containing 90% of colloidal silica and 10% of hydrogen peroxide. The microstructure of different zones was examined using orientation imaging microscopy (OIM). The OIM characterization was carried out by electron back scatter diffraction pattern using a Philips ESEM-XL30 scanning electron microscope (SEM) equipped with a field emission gun operating at 20 kV. Nanoindentation tests were performed using MTS XP Nano-indenter machine, equipped with a Berkovich indenter. Ten indentations were conducted for each weld zone at the constant maximum load of 30 mN.

**Figure 1:**
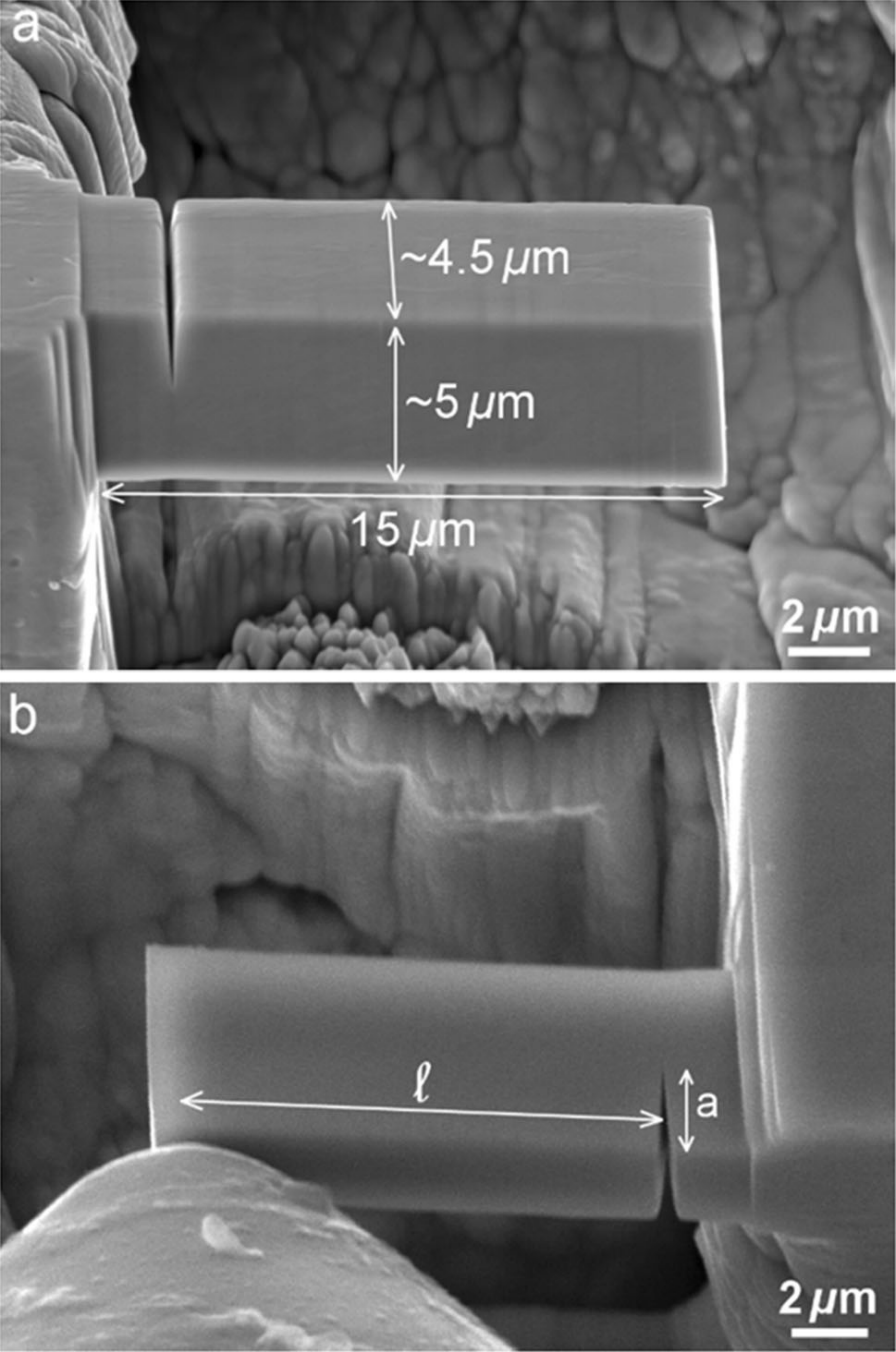
**a** Representative micrograph of FIB-milled cantilever with a notch and **b** in situ microcantilever bending overview (the sample is tilted 25° with respect to the electron beam)

Milling of cantilevers in selected regions of DP1000-GI welds was conducted on Tescan LYRA SEM–FIB dual beam microscope. The cantilever beams with a nominal length of 15 µm, a thickness of 5–5.5 µm and a width of 4–4.5 µm were roughly milled using higher current Ga^+^-ion beam (10 nA, 3 nA and 600 pA) and polished using lower current (200 pA and 40 pA) at 30 kV (Fig. 1). The cantilevers were notched using a low ion beam current of 10 pA to produce sharp notch as much as possible with the crack length to width (*a*/*t*) ratio of 0.35–0.4. The bending of cantilevers was performed in the dual beam microscope by a nanotester (ASMEC, Germany) under the displacement-controlled mode equipped with a spherical diamond indenter. This minimizes possible impression by a sharp indenter on the cantilever, and therefore, the measured displacement is primarily the deflection of the cantilever rather than local deformation in the contact area. Several loading and unloading steps with the rate of 20 nm/s were applied to monitor the crack propagation during bending.

## Results and discussion

### Microstructure

**Figure 2:**
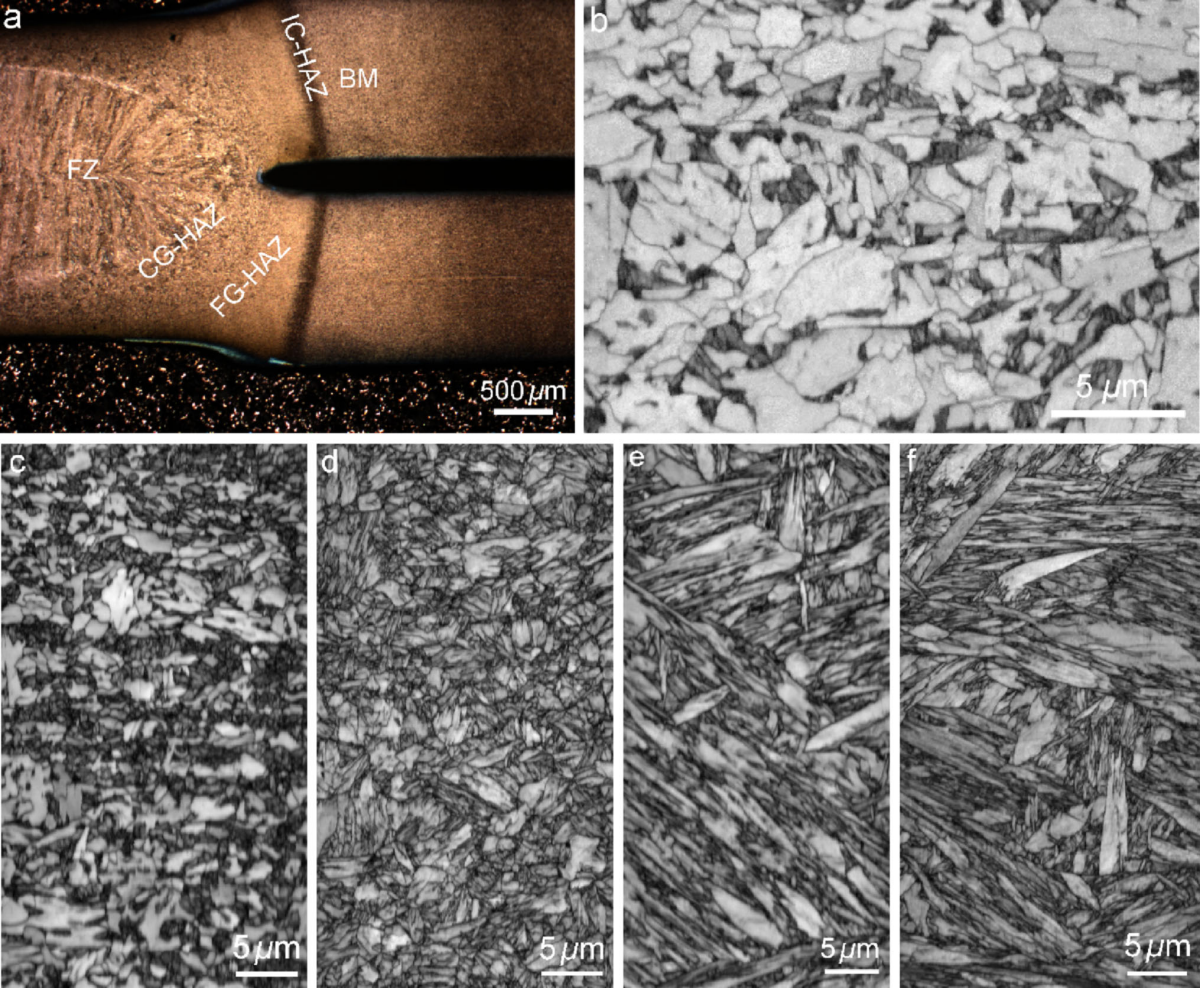
**a** Cross section of the resistance spot weld showing different weld zones. Image quality map of OIM showing the microstructure of the BM (**b**), IC-HAZ (**c**), FG-HAZ (**d**), CG-HAZ (**e**) and FZ (**f**)

Figure 2a shows an optical microscopy image of the weld cross section together with labels for different weld zones. Image quality (IQ) map of each zone is shown in Fig. 2b–f. BM consists of dual-phase structure of ferrite and martensite. Because of higher dislocation density and lattice distortion, martensite shows lower IQ and appears darker in the image, which enables separation of ferrite from martensite (Fig. 2b). The peak temperature in the IC-HAZ ranges between Ac_1_ and Ac_3_. The increase in the peak temperature within this range results in an increase in the volume fraction of ferrite dissolved into the austenite. Subsequent rapid cooling induced by the electrodes leads to the transformation of inter-critically austenite phase back to dual martensite-ferrite phase (Fig. 2c). As illustrated the volume fraction of martensite phase is higher in this zone compared to BM. FG-HAZ often lies in the area with partial or full transformation but little grain growth. As shown in Fig. 2d, this area is composed of ultra-fine martensite combined with small fraction of untransformed ferrite. The average block thickness of martensite in this zone is 515 nm. The peak temperature in the CG-HAZ exceeds well above the Ac_3_ temperature, leading to the formation of fully austenitized microstructure. The CG-HAZ is adjacent to the weld nugget, which facilitates grain growth. Subsequent rapid cooling transforms CG-HAZ to coarser martensitic microstructures with an average block thickness of 960 nm (Fig. 2e). Fusion zone (FZ) is the zone which is melted and resolidified during the welding leading to the formation of elongated blocks of martensite inside the columnar structure of prior austenite grains (Fig. 2f). The average block thickness in the FZ was measured as 1.1 µm.

### Mechanical properties

**Figure 3:**
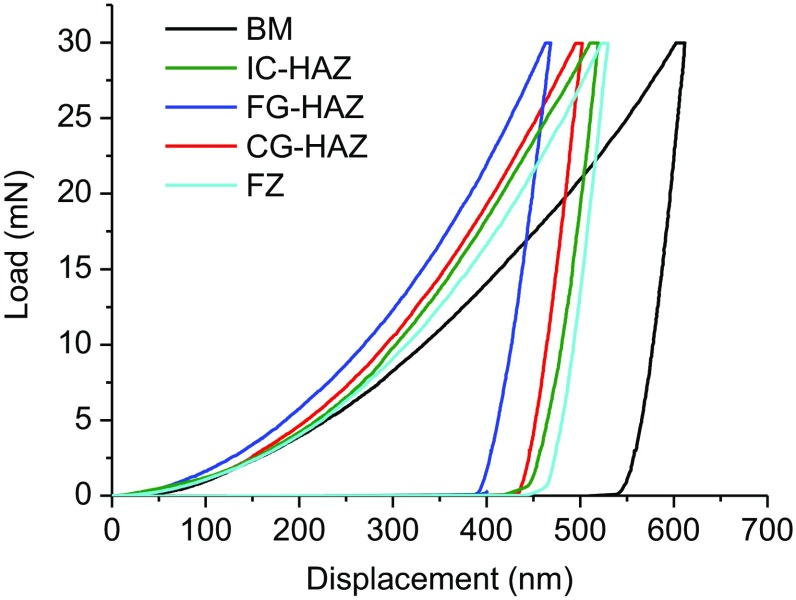
Load–displacement curves of nanoindentation test for different weld zones

Nanoindentation experiments were performed to assess local yield strength of material at different weld zones. To minimize the effect of nanoindentation size and the inhomogeneity of multiphase materials such as the DP1000-GI dual-phase high-strength steel, special attention was made in the selection of the maximum load for nanoindentation, which had to be large enough to ensure that the indentation included both ferrite and martensite phases at the different weld zones. In rare cases only martensite or ferrite was indented leading to very high or low hardness value, these indentation data were identified by means of SEM inspection on the indentations and excluded from the measurement. The representative load–displacement curves for the five zones are shown in Fig. 3. The average hardness and Young’s moduli values are listed in Table 1. As illustrated, the BM and FG-HAZ have the lowest and highest hardness values, respectively. As expected, similar moduli E were measured for different weld zones as their structure is almost martensitic. A relatively lower value of E was obtained for the BM with dual-phase structure of ferrite and martensite. The IC-HAZ also has a comparable E value as that of the FG-HAZ, CG-HAZ and FZ.

**Table 1:** Hardness and elastic modulus of weld zones obtained using nanoindentation test

	*H* (GPa)	*E* (GPa)
BM	3.9 ± 0.13	219 ± 7.5
IC-HAZ	5.2 ± 0.28	228 ± 19.6
FG-HAZ	6.3 ± 0.25	239 ± 6.8
CG-HAZ	5.6 ± 0.27	226 ± 13.6
FZ	5.1 ± 0.11	234 ± 15.4

**Figure 4:**
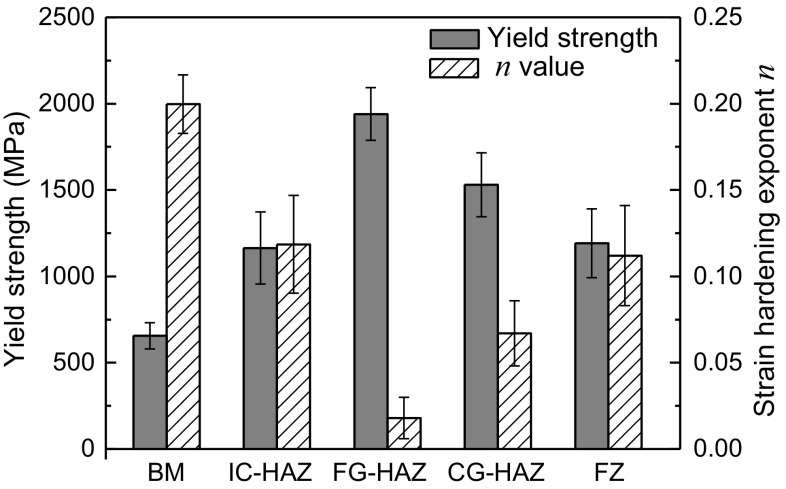
Extracted yield strength and strain hardening exponent from nanoindentation tests

To extract yield strength and strain hardening exponent from nanoindentation data, dimensional analysis developed by Dao et al. [18] was used. They established a forward and reverse algorithm to determine analytical solutions to relate indentation data to elasto-plastic properties of ductile materials as presented in the appendix. The extracted *σ*_*y*_ and *n* values for different weld zones are depicted in Fig. 4. The BM shows the highest average *n* value of 0.2 and the lowest *σ*_*y*_ of 656 MPa, which is in good agreement with the yield strength (~ 700 MPa at 0.2% offset) of DP1000-GI steel measured with standard tensile test. The FG-HAZ achieves the highest *σ*_*y*_ of 1940 MPa and the lowest *n* value of 0.018. This is attributed to the ultra-fine fully martensitic structure of FG-HAZ. Decrease in block thickness of martensite as the mean free path of dislocations leads to a higher yield strength, but it diminishes the capacity of material to work harden after yielding. While the second highest *σ*_*y*_ and the second lowest *n* value are measured in CG-HAZ, IC-HAZ and FZ show almost the same range of yield strength and n values despite their different microstructures. Such a large difference between the mechanical properties of BM and entire HAZ was also reported by Tong et al. [8].

### Fracture toughness

**Figure 5:**
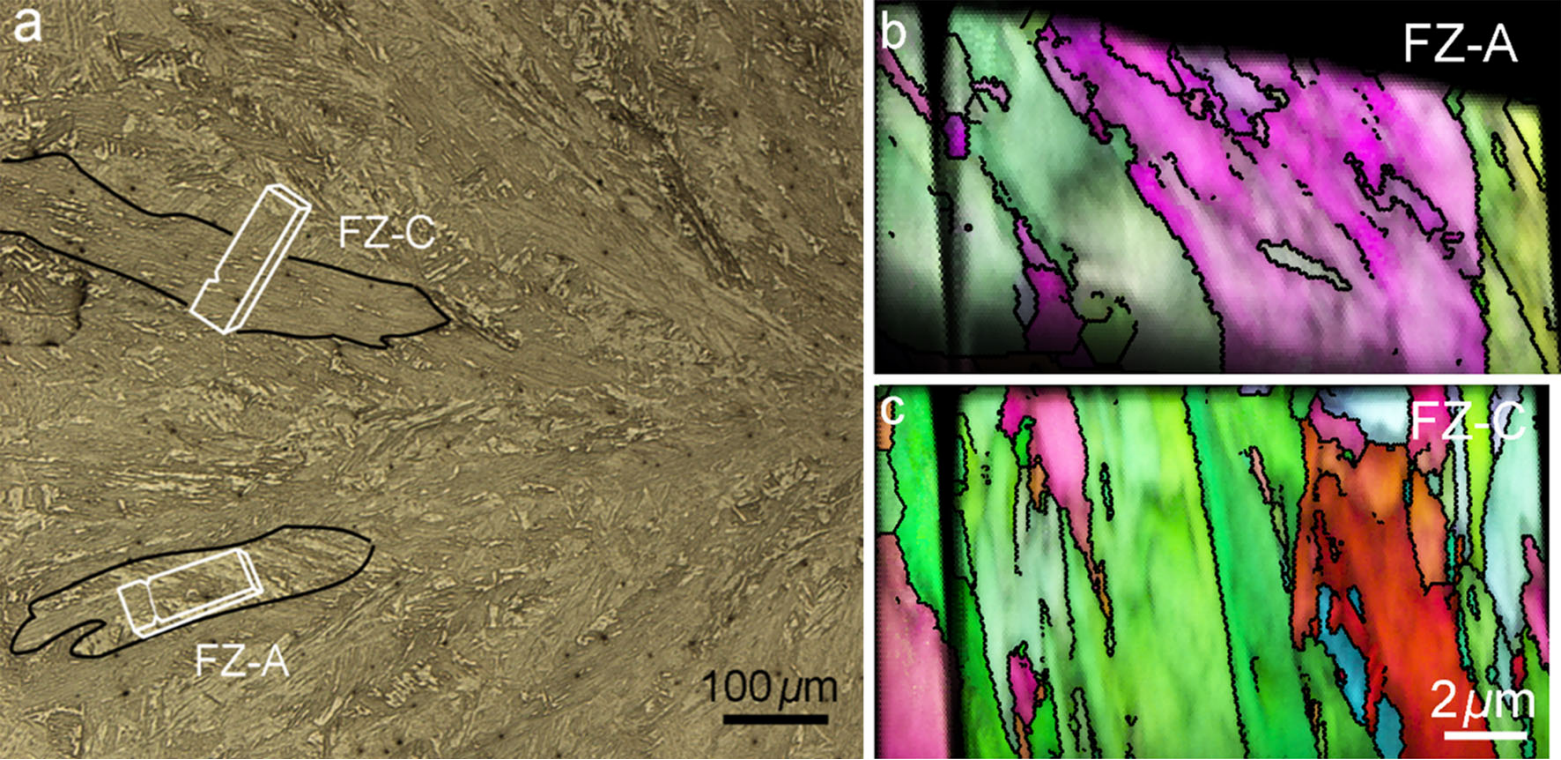
Overview of the FZ together with schematic image of milled cantilevers in two directions (**a**), inverse pole figure map from top side of cantilever milled along (**b**) and across (**c**) the columnar structure of prior austenite grain.(Prior austenite grain boundaries in (**a**) and block boundaries in (**b**) and (**c**) are highlighted with black lines. Cantilever sizes have been drawn exaggeratedly in (**a**) for better indication)

Local fracture properties of ductile materials cannot be identified through indentation methods as they are based on the measurement of crack length at indentation corners that were originally applied for brittle materials [19]. Therefore, micro-sized cantilever testing seems to be the most feasible method to evaluate the fracture properties of weld zones. Micro-cantilevers were milled in different weld zones as described in “Experimental” section. For the FZ two cantilevers were milled in two different directions as schematically shown in Fig. 5a. In this map the columnar grain boundaries of two prior austenite grains are shown as black lines. The inverse pole figure map from the top side of the two cantilevers is shown in Fig. 5b, c, respectively. Block boundaries are highlighted by black lines. The cantilever labeled as FZ-A was fabricated along the columnar grain in which the block boundaries cross over the notch and make an angle around 45° with it (Fig. 5b). The second one named as FZ-C was milled across the columnar grain in which the block boundaries are almost parallel to the notch (Fig. 5c).

**Figure 6:**
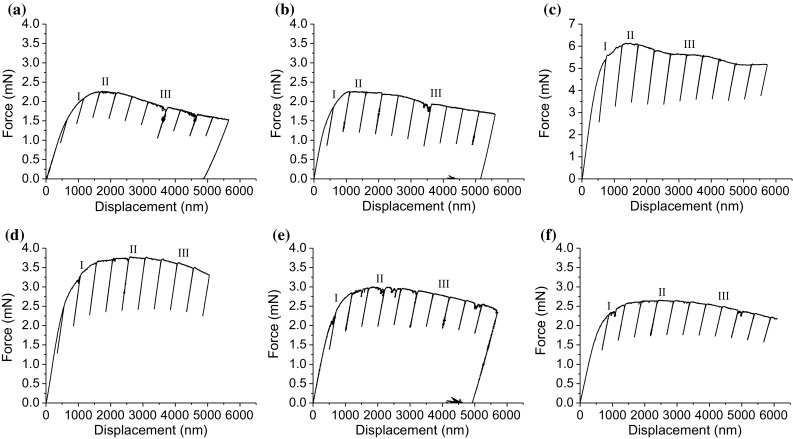
Load–displacement curve of the BM (**a**), IC-HAZ (**b**), FG-HAZ (**c**), CG-HAZ (**d**), FZ-A (**e**), FZ-C (**f**)

Figure 6 shows the experimental load–displacement curves for bending the micro-cantilevers of the different weld zones. Partial unloading segments were used after a specific displacement interval (500 nm). This enables to determine the stiffness of the cantilever by each unloading segment for tracking crack propagation. Three different stages are observed for the bending of all cantilevers. The first stage (I) is associated with yielding and strain hardening which shows an increase in load with displacement. Stage (II) is achieved by a force plateau during which the change in load with displacement is insignificant. The force plateau is followed by stage (III) that shows a continuous decrease in load with increasing displacement. As observed, all the cantilevers show large plastic deformations during loading that make the linear-elastic fracture mechanics (LEFM) inapplicable.

As not all the requirements set by the standards [20, 21] to determine the stress intensity factor (*K*_IC_) are satisfied by micro-sized cantilever testing, all the measured values of fracture toughness are termed “conditional” in this study and labeled with a subscript “Q”. The conditional stress intensity using LEFM for a notched cantilever is obtained by [14, 20]:

1 $$ K_{\text{IQ}} = \frac{{F_{\text{Q}} L}}{{{\text{wt}}^{3/2} }} f\left( {\frac{a}{t}} \right) $$

where *F*_Q_ is the force determined according to [20], *L* is the bending length between the notch and the loading point, *w* the width and *t* the thickness of micro-cantilever (see Fig. 1). Dimensionless shape factor *f*(*a*/*t*) for a rectangular cantilever geometry is calculated using the expression taken from [14]:

2 $$ f\left( {\frac{a}{t}} \right) = 4*\frac{{3\left( {\frac{a}{t}} \right)^{0.5} (1.23 - \left( {\frac{a}{t}} \right)\left( {1 - \left( {\frac{a}{t}} \right)} \right)\left( { - 6.09 + 13.96\left( {\frac{a}{t}} \right) - 14.05\left( {\frac{a}{t}} \right)^{2} } \right)}}{{2\left( {1 + 2\left( {\frac{a}{t}} \right)} \right)\left( {1 - \left( {\frac{a}{t}} \right)} \right)^{1.5} }} $$

If LEFM is applied to the maximum load where the crack initiates in Fig. 6, the lowest and highest *K*_IQ_ of 1.46 and 3.35 MPa m^1/2^ is extracted for the BM and FG-HAZ, accordingly. ASTM standard [21] sets restrictions for the sample dimension as the ligament size (*t* − *a*_0_) must be larger than $2.5 \left( {\frac{{K_{\text{IQ}}^{2} }}{{\sigma_{Y}^{2} }}} \right)$. According to the obtained *K*_IQ_ and yield strength, the minimum ligament size for the BM and FG-HAZ would be 12.3 and 7.45 µm, respectively, which are larger than the proposed size by the standard. LEFM can only be used when there is not large-scale yielding in front of a crack tip and thus provides the lower limit of the fracture toughness.

Therefore, other methods including *J*-integrals and crack tip opening displacement (CTOD) have been applied to evaluate the fracture toughness of semi-brittle and ductile materials, which we discuss in the following sections.

#### CTOD

**Figure 7:**
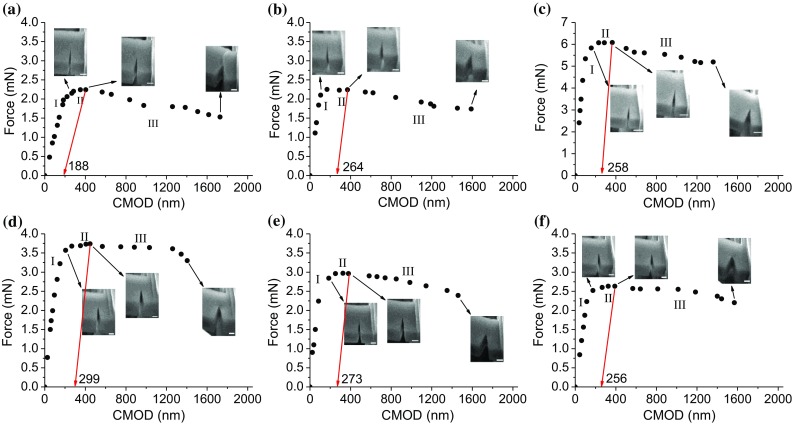
Force-CMOD curves of the BM (**a**), IC-HAZ (**b**), FG-HAZ (**c**), CG-HAZ (**d**), FZ-A (**e**) and FZ-C (**f**) cantilevers. All the scale bars in the insert SEM images are 1 µm

CTOD is one of the most widely used non-linear methods to determine the fracture toughness of ductile materials with large-scale yielding. According to the standard for macroscale samples [21], a notch or pre-crack must be created using fatigue test. It requires specific sample geometries like the arc-shaped or 3-point bending specimens. None of them are fulfilled at micro-scale testing and thus again the ‘conditional’ values are calculated. In order to determine CTOD_Q_, it is needed to measure the crack mouth opening displacement (CMOD). It was carried out by capturing multiple SEM images during the loading process of beams. Figure 7 shows the plot of bending force versus CMOD for the tested cantilevers. Three stages can be identified in the plots. Small CMOD is achieved by yielding (stage I), which is followed by a noticeable CMOD as reaching the force plateau (stage II). More pronounced CMOD is observed after the force plateau while it is accompanied by a drop in load and hence larger crack propagation (stage III). As indicated in Fig. 7c, stage II of force plateau ends with slightly smaller but still comparable CMOD for the FG-HAZ compared to other samples. However, it is associated with a much higher load.

The CTOD_Q_ can be calculated by [22]:

3 $$ {\text{CTOD}}_{\text{Q}} = \delta_{\text{Q}} = \delta_{\text{Q}}^{\text{el}} + \delta_{\text{Q}}^{\text{pl}} = d_{n} \frac{{K_{\text{Q,LEFM}}^{2} \left( {1 - \nu^{2} } \right)}}{{\sigma_{Y} E}} + \frac{{r_{\text{pl}} \left( {t - a_{0} } \right)\nu_{\text{pl}} }}{{r_{\text{pl}} \left( {t - a_{0} } \right) + a_{0} }} $$

where *d*_*n*_ is a dimensionless factor equal to 0.5 assuming plain strain condition. $\sigma_{Y}$ and *E* take the values obtained in “Mechanical properties” section via nanoindentation tests. $r_{\text{pl}}$ is the plastic rotational factor and set to 0.44 based on the hinge model for the single-edge bend geometry. $\nu_{\text{pl}}$ is the plastic part of the displacement and can be achieved by making a construction line from the end of force plateau parallel to the initial elastic loading line of force-CMOD curve. The fracture toughness *K*_IQ, *δ*_ is then calculated from:

4 $$ K_{{{\text{Q}},\delta }} = \sqrt {\frac{1}{{d_{n} }}\frac{{\sigma_{Y} E}}{{\left( {1 - \nu^{2} } \right)}}\delta_{\text{Q}} } $$

The calculated *K*_Q, δ_ values for the BM, IC-HAZ, FG-HAZ, CG-HAZ, FZ-A and FZ-C are 4.96, 7.20, 11.59, 10.05, 7.9 and 7.38 MPa m^1/2^, respectively.

#### *J*-integral

Beside the possibility to measure CTOD, *J*-integral can be used to evaluate fracture toughness of materials with large-scale yielding. This method is based on a precise knowledge of crack extension during loading. This can be achieved by measuring the beam stiffness for each unloading segment. Crack propagation leads to a reduced ligament size and thus a lowered bending stiffness. By determining the stiffness (*k*_*i*_) for each unloading segment, the change in ligament size (*t* − *a*_*i*_) can be estimated using:

5 $$ t - a_{i} = \sqrt[3]{{\frac{{4k_{i} L^{3} }}{wE}}} $$

As already discussed, the stage I is associated with yielding and strain hardening. Strain hardening occurs because of significant plasticity in front of a notch leading to high resistance against crack propagation. The unloading segments show an increase in the stiffness before reaching the maximum load. It was assumed that no crack prorogation occurs during strain hardening and unloading segments before reaching the maximum load were excluded for the sake of determining the turning point of stiffness evolution that corresponds to crack propagation. In stage II a force plateau is reached as two factors in completion: strain hardening and blunting of newly formed crack tip tends to increase the load, whereas crack propagation leads to a smaller beam cross section and decreases the required load for further deformation. The FG-HAZ shows more limited strain hardening and force plateau. In contrast, in the case of the CG-HAZ, FZ-A and FZ-C, it takes larger displacement to overcome the stage II. The third stage (III) is characterized by continuous decrease in load and bending stiffness that is because of stable crack growth, which completely overcomes the strain hardening.

**Figure 8:**
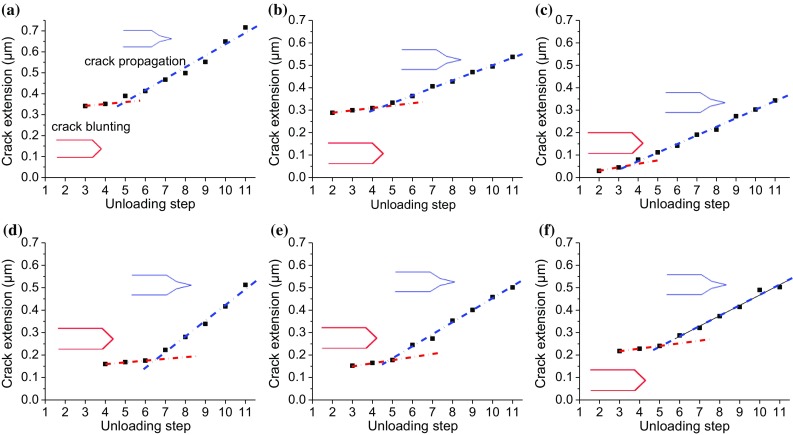
Crack extension versus unloading step for the BM (**a**), IC-HAZ (**b**), FG-HAZ (**c**), CG-HAZ (**d**), FZ-A (**e**) and FZ-C (**f**)

Figure 8 illustrates the plot of the estimated crack extension by each step of unloading. Two distinct stages of crack extension for all the samples can be identified. The first stage is called crack blunting during which the crack growth rate is slow. This stage corresponds to the stage II of force plateau in the force–displacement curves in Figs. 6 and 7. During the second stage, sharp crack propagation with higher growth rate occurs. It corresponds to the stage III that is associated with stable crack growth and continuous decrease in load presented in Figs. 6 and 7. Clearly, crack blunting is less effective in the FG-HAZ as the transition to the second stage of crack propagation occurs after the third unloading segment. The crack blunting effect is the strongest in the case of the CG-HAZ as the transition to the second stage is delayed after the 6th unloading step. However, the smallest crack extension is observed for the FG-HAZ as opposed to the BM with the largest crack length. It should be also considered that crack propagation occurs at much higher loads for the FG-HAZ sample.

The *J*-integral of *i*th unloading segment can be calculated using [21]:

6 $$ J_{{{\text{Q}},i}} = J_{i}^{\text{el}} + J_{i}^{\text{pl}} = \frac{{\left( {K_{\text{IQ}} } \right)^{2} \left( {1 - \nu^{2} } \right)}}{E} + \left[ {J_{i - 1}^{\text{pl}} + \frac{{\eta \left( {A_{i}^{\text{pl}} - A_{{\left( {i - 1} \right)}}^{\text{pl}} } \right)}}{{w\left( {t - a_{{\left( {i - 1} \right)}} } \right)}}} \right]\left[ {1 - \frac{{a_{i} - a_{{\left( {i - 1} \right)}} }}{{\left( {t - a_{{\left( {i - 1} \right)}} } \right)}}} \right] $$

The *J*-integral is split into two parts, namely elastic and plastic part. The elastic part is calculated using *K*_IQ_ that is obtained this time by setting *F*_Q_ = *F*_0.95_ in the ith unloading part. *F*_0.95_ is the load obtained by making a construction line with 95% of the slope of the reloading part of every unloading segment. In the plastic part, *η* is a constant and equals to 2 [14], *A*^pl^ represents the area beneath the load–displacement curve excluding the triangle part defined by the *i*th unloading line. Once the *J*_Q_ is extracted from *J* curve versus crack extension, the conditional fracture toughness can be achieved by:

7 $$ K_{{{\text{Q}},J}} = \sqrt {\frac{{J_{\text{Q}} E}}{{1 - \nu^{2} }}} $$

**Figure 9:**
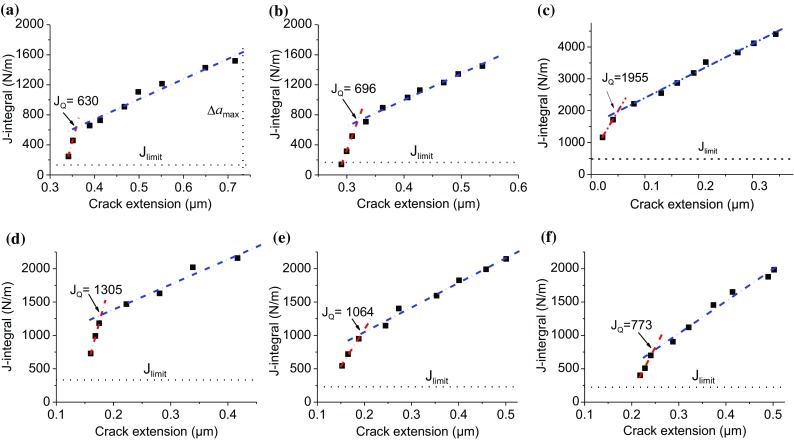
*J*-integral versus crack extension (*J* − Δ*a*) curves of the BM (**a**), IC-HAZ (**b**), FG-HAZ (**c**), CG-HAZ (**d**), FZ-A (**e**) and FZ-C (**f**)

Figure 9 shows *J* − Δ*a* curves for different cantilevers. All the curves exhibit typical shape as observed for the ductile materials tested at macroscale with a blunting line followed by stable crack growth. *J* − Δ*a* curve for the stable crack growth must be fitted by the power law of the form: $J\left( {\Delta a} \right) = C_{1} \left( {\frac{\Delta a}{k}} \right)^{{C_{2} }}$, where *k* is a constant and *C*_1_ and *C*_2_ are determined by fitting procedure. In a standard test, a construction line parallel to the blunting line is drawn at the offset of 0.2 mm. The intersection of this line with the curve of stable crack growth gives *J*_Q_ value. However, it is not possible to make such a large offset at the micrometer scale used in the work.

Here we also follow Wuster et al. [14] who proposed another method to extract *J*_Q_ from the *J* − Δ*a*. It includes fitting of the data with two linear functions. The first line describes the initial part of the curve for blunting part, while the second line is made by fitting the data for the stable crack growth part. The intersection of these two lines holds an estimate for the critical *J* that indicates a transition from one stage to another. The standard test restricts the maximum *J* value and crack propagation to gain a valid value for the fracture toughness. The limitation for *J*-integral and crack extension is given by $J_{\text{limit}} = \sigma_{Y} \frac{{t - a_{0} }}{15}$ and $\Delta a_{\text{limit}} = 0.25\left( {t - a_{0} } \right)$, respectively [23].

The dashed lines in Fig. 9a show these limitations for *J* value and crack extension for the BM. As observed, *J* value limitations are not met as all the measured values are above the *J*_limit_. In contrast, the measured values of the crack extension are below the maximum crack propagation Δ*a*_max_ allowed by the standard. The same conditions hold for all other samples as the *J*_limit_ requirements are not fulfilled, whereas the crack extension is smaller than Δ*a*_max_. However, as Δ*a*_max_ values (≥ 0.7 µm) for other samples are far above the measured crack extension, they have not been shown in the graphs.

**Figure 10:**
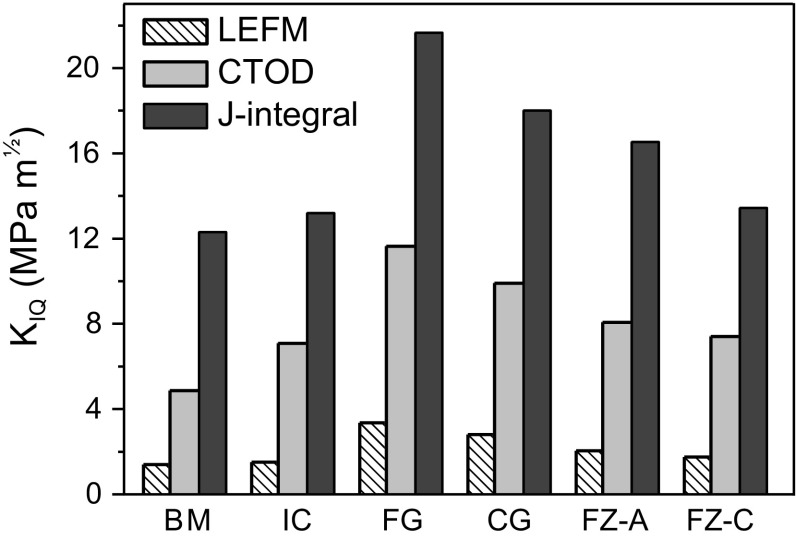
Measured conditional fracture toughness values using LEFM, CTOD and *J*-integral methods

The conditional fracture toughness values measured using the three methods, namely LEFM, CTOD and *J*-integral are shown in Fig. 10. LEFM only provides the lower bound of the fracture toughness for ductile materials. CTOD method yields lower fracture toughness compared to *J*-integral. It can be attributed to the sample size effect as there is an increase in the yield strength with decrease in sample size [24]. Demir et al. [25] studied micro-cantilever bending of single crystalline copper and reported higher flow stress for smaller beams. Increase in the yield strength due to size effect decreases the required sample size, but on the other hand increases the fracture toughness measured through CTOD method. In the present study, the yield strength of different weld zones was extracted from nanoindentation test at which the effect of sample size is effective, especially when the depth of penetration is small. By taking into account this issue, the selected maximum load for nanoindentation was high enough to yield a large indentation depth, which makes the scale dependent effects negligible. This is reflected also in the obtained yield strength for the BM, which is in consistency with the yield strength measured at macroscale. As the incorporated yield strength obtained from nanoindentation might be lower than the real yield strength of the structure in front of the notch, the CTOD method leads to lower fracture toughness value compared to the *J*-integral. The rise in yield strength at micro-scale can be attributed to the limited amount of active dislocation sources in small volume or to the dislocation pile up at the center of cantilever. Therefore, *J*-integral method results in more realistic values compared to CTOD method in this particular case. Nevertheless, the trend of measured fracture toughness using CTOD method is consistent with the values obtained from *J*-integral.

As indicated the FG-HAZ yields the highest fracture toughness using both CTOD and *J*-integral methods. It can be ascribed to the ultra-fine structure of the FG-HAZ. It was already reported that the refinement of martensite can effectively enhance its fracture toughness [26–28]. Packet and block boundaries are effective barrier against crack propagation that leads to higher energy for the crack to cross over the boundary. It should be noted that the maximum crack extension for all the samples is smaller than their corresponding transition flaw size ($$ a_{t} = {\raise0.7ex\hbox{${K_{\text{IQ}}^{2} }$} \!\mathord{\left/ {\vphantom {{K_{\text{IQ}}^{2} } {\pi \sigma_{Y}^{2} }}}\right.\kern-0pt} \!\lower0.7ex\hbox{${\pi \sigma_{Y}^{2} }$}} $$). Therefore, the failure of the welds is governed by plastic yielding (otherwise at crack sizes larger than *a*_*t*_, the failure would be dominated by fracture mechanics). For the FG-HAZ with higher yield strength, higher energy is consumed at the crack tip to create new surfaces for crack propagation.

**Figure 11:**
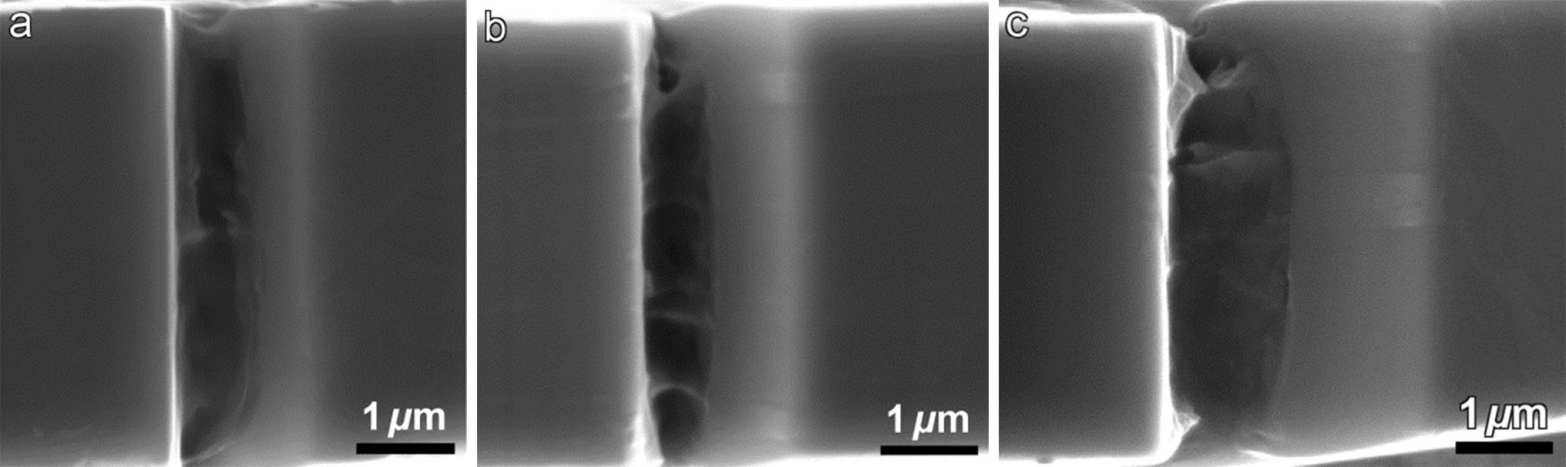
Fracture surface of bended cantilevers for the FG-HAZ (**a**), FZ-A (**b**) and FZ-C (**c**)

Figure 11 shows representative fracture surface of bended cantilevers for the FG-HAZ, FZ-A and FZ-C. As indicated, samples fail in a ductile manner as the fracture is associated with the formation of micro-voids and dimples. Therefore, it can be deduced that micro-cantilevers yield before fracture. It is also worth noting that more homogeneous fracture surface is observed for the FZ-C. It can be because of alignment of the notch along the block boundaries that makes the delamination of structure and crack propagation easier. It is also reflected in the measured fracture toughness, as the FZ-C shows larger crack propagation and lower fracture toughness compared to FZ-A.

The measured values of the fracture toughness of weld zones at micro-scale are lower than the reported values for martensitic steels. It can be attributed to the fact that the crack extension is small at micro-scale and not all the toughening mechanisms such as crack deflection and crack bridging are activated. Therefore, micro-cantilever bending tests measure the toughness values for the crack initiation stage, which may increase through a larger crack propagation [29]. Nevertheless, the method provides insight about the fracture behavior of different zones in RSW for comparative study. It can be used to correlate between welding parameters, microstructure and mechanical performance.

## Conclusion

Local mechanical properties of different weld zones of DP1000-GI resistance spot weld were evaluated using nanoindentation and micro-cantilever bending tests. The yield strength and strain hardening exponent were derived from nanoindentation tests. FIB made notched micro-cantilevers were used to measure the fracture toughness of different weld zones. It is shown that the bending of cantilevers is associated with large plastic yielding, which makes the linear-elastic fracture mechanics inapplicable.

Cyclic loading can be applied to measure the fracture toughness at micro-scale using *J*-integral and crack tip opening displacement methods. It enables tracking crack extension by measuring the beam stiffness at each unloading segment. The measured values are lower than the fracture toughness of macro-sized samples. However, the method can be successfully implemented to the investigation of resistance spot welds for comparative study. It paves the way to make detailed and accurate correlation between welding parameters and mechanical performance in order to develop a model for the prediction of mechanical properties of resistance spot welds.

## Appendix

Estimation of yield strength and strain hardening exponent (*n*) from nanoindentation tests.

Assuming that plastic behavior of the material can be approximated by a power law description, the nominal stress *σ* can be defined by:

A1 $$ \sigma = \sigma_{y} \left( {1 + \frac{E}{{\sigma_{y} }}\varepsilon_{p} } \right)^{n} $$

The stress at the plastic strain ($\varepsilon_{p}$) of 0.033 is described by:

A2 $$ \frac{C}{{\sigma_{0.033} }} = - 1.131\left[ {\ln \left( {\frac{{E^{*} }}{{\sigma_{0.033} }}} \right)} \right]^{3} + 13.635\left[ {\ln \left( {\frac{{E^{*} }}{{\sigma_{0.033} }}} \right)} \right]^{2} - 30.594\left[ {\ln \left( {\frac{{E^{*} }}{{\sigma_{0.033} }}} \right)} \right] + 29.267 $$

where *C* is the curvature of the loading curve and is defined by:

A3 $$ P = Ch^{2} $$

*E** is the reduced Young modulus and is given by:

A4 $$ E^{*} = \frac{1}{2.12}\frac{\sqrt \pi }{\sqrt A }\frac{{{\text{d}}P_{u} }}{{{\text{d}}h}}|_{{h_{m} }} $$

For a Berkovich indenter the contact area *A* is equal to $24.56h_{m}^{2}$. By determining the slope of unloading at maximum load-depth, $\frac{{{\text{d}}P_{u} }}{{{\text{d}}h}}|_{{h_{m} }}$, strain hardening exponent can be calculated using:

A5 $$ \begin{aligned} & \frac{1}{{E^{*} h_{m} }}\frac{{{\text{d}}P_{u} }}{{{\text{d}}h}}|_{{h_{m} }} = \left( { - 1.40557n^{3} + 0.77526n^{2} + 0.1583n - 0.06831} \right)\left[ {\ln \left( {\frac{{E^{*} }}{{\sigma_{0.033} }}} \right)} \right)^{3} + (17.93006n^{3} - 9.22091n^{2} - 2.37733n + 0.86295) \\   & \left[ {\ln \left( {\frac{{E^{*} }}{{\sigma_{0.033} }}} \right)} \right]^{2} + \left( { - 79.99715n^{3} + 40.5562n^{2} + 9.00157n - 2.54543} \right)\left[ {\ln \left( {\frac{{E^{*} }}{{\sigma_{0.033} }}} \right)} \right] + \left( {122.65069n^{3} - 63.88418n^{2} - 9.58936n + 6.20045} \right) \\ \end{aligned} $$

The yield strength can be obtained by:

A6 $$ \sigma_{0.033} = \sigma_{y} \left( {1 + \frac{E}{{\sigma_{y} }}0.033} \right)^{n} $$

Representative $\varepsilon_{0.033}$ was selected as it was found that dimensionless function of (A2) normalized with respect to $\sigma_{0.033}$ is independent of strain hardening exponent *n*.
